# Equitable access to quality injury care; Equi-Injury project protocol for prioritizing interventions in four low- or middle-income countries: a mixed method study

**DOI:** 10.1186/s12913-024-10668-y

**Published:** 2024-04-04

**Authors:** Equi-Injury Group, Justine Davies, Kathryn Chu, Stephen Tabiri, Jean Claude Byiringiro, Abebe Bekele, Junaid Razzak, Lucia D’Ambruoso, Agnieszka Ignatowicz, Laura Bojke, Lungiswa Nkonki, Christina Laurenzi, Alice Sitch, Irene Bagahirwa, Antonio Belli, Napoleon Bellua Sam, Alemayehu Amberbir, John Whitaker, Denys Ndangurura, Leila Ghalichi, Tamlyn MacQuene, Ntombekhaya Tshabalala, Derbew Fikadu Berhe, Ntezimana Jean Nepomuscene, Anita Eseenam Agbeko, Frederick Sarfo-Antwi, Zaheer Babar Chand, Zabin Wajidali, Fazila Sahibjan, Huba Atiq, Yonela Mali, Zola Tshabalala, Faieeza Khalfe, Olwethu Nodo, Ghislaine Umwali, Eric Twizeyimana, Nadine Mugisha, Ngirabeza Oda Munyura, Solange Nakure, Sage Marie Consolatrice Ishimwe, Pascal Nzasabimana, Adams Dramani, Jane Acquaye, Ahmed Tanweer

**Affiliations:** Institute of Applied Health Research, University of Birmingham, Birmingham, UK

**Keywords:** Equity, LMIC, Injury, Mixed method

## Abstract

**Background:**

Equitable access to quality care after injury is an essential step for improved health outcomes in low- and middle-income countries (LMICs). We introduce the Equi-Injury project, in which we will use integrated frameworks to understand how to improve equitable access to quality care after injury in four LMICs: Ghana, Pakistan, Rwanda and South Africa.

**Methods:**

This project has 5 work packages (WPs) as well as essential cross-cutting pillars of community engagement, capacity building and cross-country learning. In WP1, we will identify needs, barriers, and facilitators to impactful stakeholder engagement in developing and prioritising policy solutions. In WP2, we will collect data on patient care and outcomes after injuries. In WP3, we will develop an injury pathway model to understand which elements in the pathway of injury response, care and treatment have the biggest impact on health and economic outcomes. In WP4, we will work with stakeholders to gain consensus on solutions to address identified issues; these solutions will be implemented and tested in future research. In WP5, in order to ascertain where learning is transferable across contexts, we will identify which outcomes are shared across countries. The study has received approval from ethical review boards (ERBs) of all partner countries in South Africa, Rwanda, Ghana, Pakistan and the University of Birmingham.

**Discussion:**

This health system evaluation project aims to provide a deeper understanding of injury care and develop evidence-based interventions within and across partner countries in four diverse LMICs. Strong partnership with multiple stakeholders will facilitate utilisation of the results for the co-development of sustainable interventions.

**Supplementary Information:**

The online version contains supplementary material available at 10.1186/s12913-024-10668-y.

## Background

Injuries are predicted to be the third leading cause of death by 2030 [1]. They already cause 9% of global mortality [2, 3]. Reducing trauma related mortality worldwide could save 2 million lives per year, with an associated economic benefit of $245–261 billion using a human capital approach [4]. Non-fatal injuries also represent an enormous burden, with 1 billion people who sustain an injury requiring care annually and up to 40% of injured individuals reporting disability after injury [5–7]. Low- and middle-income countries (LMICs) account for 90% of deaths after injury, and cases are likely to increase along with economic development, reflecting higher motorised transport due to rising standards of living [2].

Equitable access to quality healthcare services after an injury can reduce the mortality and morbidity after injury [8–13]. A whole health system approach which provides care for the injured patient from the time a person is injured and continues until the patient is optimally rehabilitated is needed to reduce deaths and disability. However, limited systematic understanding of barriers to equitable access to quality healthcare from the point of injury to optimal rehabilitation, and hence where to intervene to ensure optimal use of resources, hinders the development of injury healthcare systems in LMICs [10, 14].

In our group’s previous work (Equi-Trauma) [15], equitable access to quality care for injured people was conceptualised using a novel combination of three previously established frameworks: the Three Delays Framework [14], the Institute of Medicine’s (IoM) framework for quality healthcare [16], and the WHO’s health systems building blocks [17]. Our findings highlighted the multiple barriers to accessing quality care that are not shared across countries, and a need for more rigorous mixed method evaluations to propose necessary interventions and policies [18]. In addition, Equi-Trauma established key relationships (contacts) with government stakeholders to ensure interest in improving equitable access to injury care.

Equi-Injury is the next step in addressing this need. Equi-Injury will be conducted in four LMICs (Ghana, Pakistan, Rwanda and South Africa). Building on preliminary work in Equi-Trauma, Equi-Injury will assess the health system using integrated frameworks and develop an injury pathway model showing how injured patients move through health systems in each country and where investments might improve outcomes in resource-constrained settings. We will work with stakeholders in each setting to develop a common understanding of their desires and needs and to co-develop solutions to improve equitable access to quality injury care. Finally, we will compare results across countries to identify shared findings, which might be transferable to other contexts. This protocol paper describes the conceptual frameworks and work packages of the Equi-Injury project.

## Context of four countries

This study will take place in Rwanda, Ghana, Pakistan, and South Africa, four LMICs with diverse social, economic, geographic, cultural, and health system contexts (see Table 1).

**Table 1:**
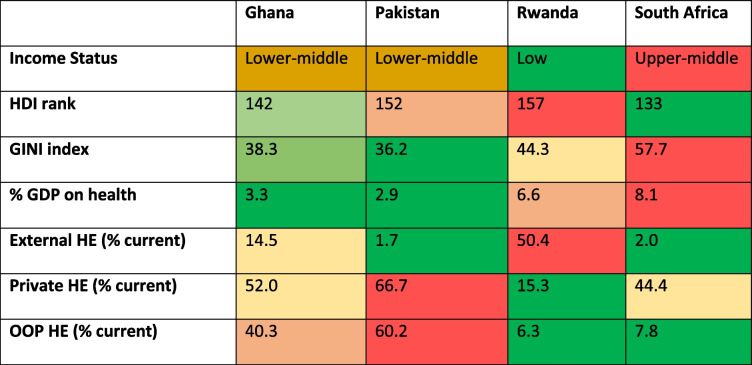
Partner countries’ economic and health expenditure profile at the time of study design, based on information from the World Bank [19]

*HE* Health expenditure, *HDI* Human Development Index, *OOP* Out of Pocket, *GDP* Gross Domestic Product. Colouring for each variable shows differences between countries (red represents the highest numerical value and green the lowest, for each variable)

Development, geographical, health systems, cultural, and injury contexts.

It is estimated that trauma accounts for 8% of deaths and 7% of DALYs in Ghana [20]. To precisely ascertain its prevalence, however, not much empirical study has been done. Ghana has a high rate of out-of-pocket (OOP) health expenditure, which mostly affects the lowest quintile of income earners who have difficulty receiving trauma care [21].

In Pakistan, injuries are estimated to cause 7% of deaths. The country has the highest OOP expenditure for health of the four study countries and there is no Universal health Coverage (UHC) in place [20, 22]. The government of Pakistan has committed to UHC and an essential package of health services has been approved.

In Rwanda, 10% of DALYs and 9% of all deaths are due to trauma. The government of Rwanda has introduced a health insurance scheme to cover services for the entire population, with contributions that are means-assessed. Although OOP payments for healthcare are decreasing, lack of enforcement of insurance policy payment and lack of granularity in the means assessments means that people who are not poor enough to be covered by the government plan, but are too poor to afford private insurance, face delays in accessing healthcare [23].

South Africa has a very high rate of trauma rate, estimated to cause to 13% of deaths, mainly from interpersonal violence [24]. Despite high proportion of the population being uninsured and dependent on the public health system, the majority of health care resources are in private sector [25].

## Conceptual background

- A. The four delays approach

The Three Delays Model (delays in seeking, reaching, and receiving care) was originally proposed by Thaddeus and Maine in 1994, conceptualising delays that contribute to maternal mortality in low-resource settings [26]. We use an expanded, Four Delay, framework that adds remaining in quality care, until being rehabilitated to optimal function, as a fourth delay which can contribute to suboptimal outcomes after injury [27]. Our definition of delays stages is as detailed in the Table 2.

**Table 2:** Description of four delays in the Four Delay approach

Delay stage	Description
**Delay 1 (seeking care)**	From the point of injury to taking the decision to seek care
**Delay 2 (reaching care)**	From deciding to seek care until reaching the facility of definitive care
**Delay 3 (receiving care)**	From arriving at the appropriate healthcare facility to receiving quality care
**Delay 4 (remaining in care)**	From discharge to rehabilitation to optimal function

Our previous work details potential barriers at each of these delays and shows that these are closely interlinked, overlapping, and reinforcing [8, 13, 26, 27]. To improve patient outcomes requires that barriers occurring at all delays are identified to understand which to address for maximal impact across the whole patient healthcare journey. Such an approach has been successful in high-income countries (HICs), resulting in a 15–25% decrease in mortality [28–31].

- B. Institute of Medicine’s (IoM) quality health framework

In this framework, quality care is conceptualised as being safe, effective, patient-centred, timely, efficient, and equitable. Where safe is defined as “avoiding injuries to patients from the care that is intended to help them”, effective is defined as “providing services based on scientific knowledge to all who could benefit and refraining from providing services to those not likely to benefit”, patient-centred is defined as “providing care that is respectful of and responsive to individual patient preferences, needs, and values and ensuring that patient values guide all clinical decisions”, timely is defined as “reducing waits and sometimes harmful delays for both those who receive and those who give care”, efficient is “avoiding waste, including waste of equipment, supplies, ideas, and energy.” and equitable is defined as “providing care that does not vary in quality because of personal characteristics such as gender, ethnicity, geographic location, and socioeconomic status” [16]. For the purposes of this study, we do not consider efficiency, as this is beyond the remit of our study.

- C. The WHO health systems building blocks

The WHO conceptualise health systems as comprised of six distinct “building blocks” including health workforce, information, medicinal products and technologies, financing, and leadership and governance. These are required to be in place to ensure that equitable access to quality healthcare is achievable [17]. The concept has widely been used in research for its simplicity and translatability between research fields, yet its ability for analysing complex and inter-linked systems is limited. Using a holistic approach to health system could help in moving away from the segmentation imposed by this framework and recognize the interactions and understanding impacts on systems as a whole [32].

### The proposed framework

**Fig. 1:**
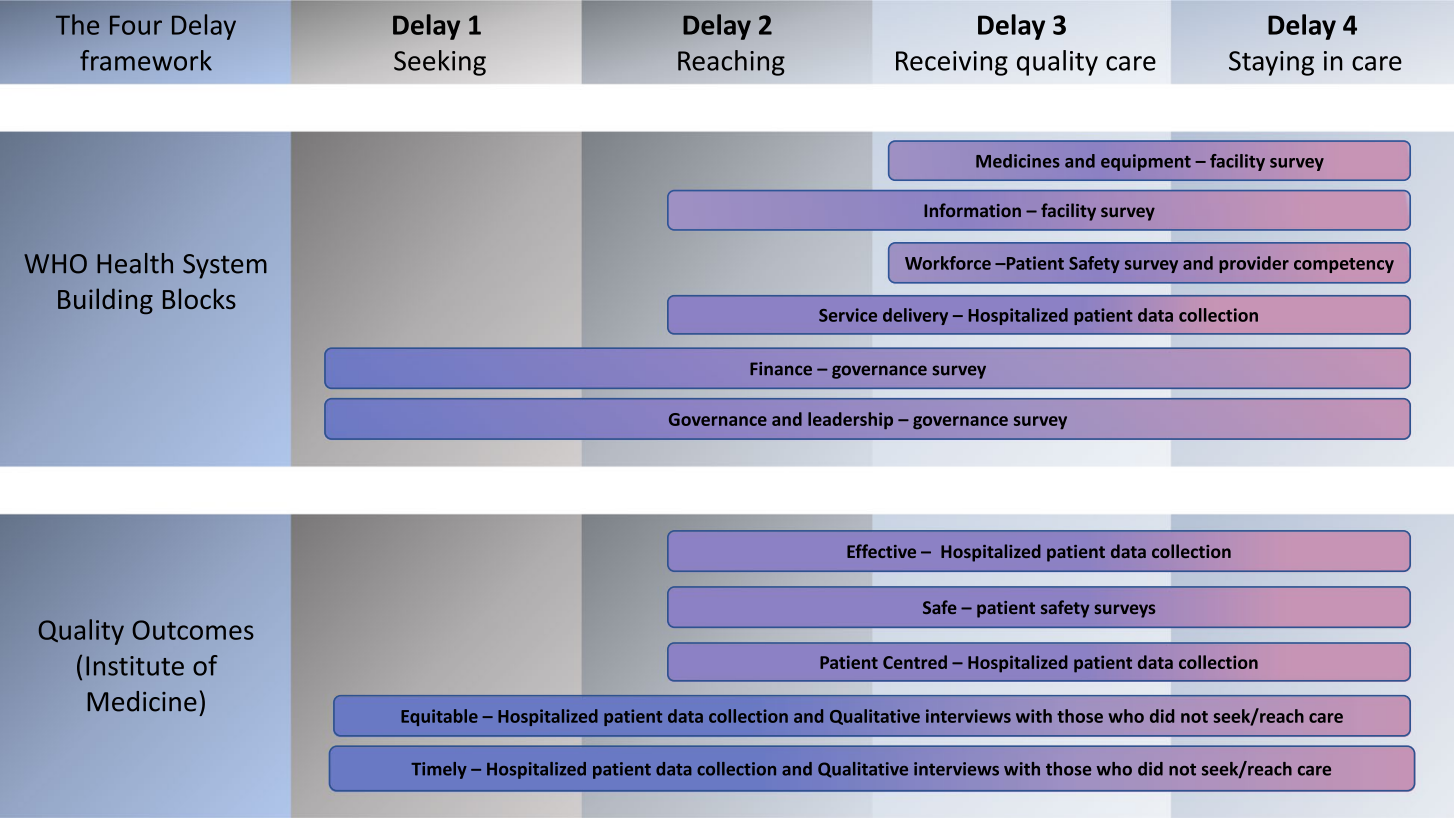
Conceptual framework for the Equi-Injury Project

We have combined the above three frameworks as demonstrated in Fig. 1. We note that the figure does not capture the complexity of the relationships between elements of each component and acknowledge that issues discovered within each of the components will interact with and potentially reinforce others. We have previously attempted to show the nature of these interactions in Rwanda [27]. In delivering Equi-Injury project, our aim is to provide evidence to further understand and summarise these complex interactions.

Figure 1 shows the components of WHO building blocks and quality outcomes evaluated in this study, and the instruments used for their evaluation and how these concepts have overlapping influences through four delays.

## Methods and analysis

The study was co-designed by the lead co-investigators in each partner country. There are five objectives and corresponding work packages (WP).

Our *objectives* are:

1. to develop a common understanding of stakeholder desires and needs to improve policy towards enabling improved equitable access to quality injury care;
2. to identify barriers and facilitators to equitable access to quality care and their consequences for the injured person;
3. to develop an injury pathway model to understand where investment is needed to promote equitable access to quality injury care which can be applied in multiple contexts;
4. to develop consensus amongst stakeholders on which solutions are priorities to address and co-develop solutions for these;
5. to synthesise cross country learning to identify similarities or differences between countries and contexts, hence where learning may be transferable elsewhere.

### Theory of change

There are three phases for the planned groups of activities. The first includes the identification of stakeholders, research, capacity building, and developing equitable partnerships. In the second, we will ascertain stakeholder needs and priorities for engagement, priorities for outcome after injury, context and governance of injury services, adverse outcomes after injury and barriers causing delays to accessing equitable care. The outcome of these activities will be used to develop strategies for stakeholder engagement and attaining shared understanding. The third group of activities consists of broad engagement with and dissemination to politicians, funders and policy groups, lay people, and the scientific community.

**Fig. 2:**
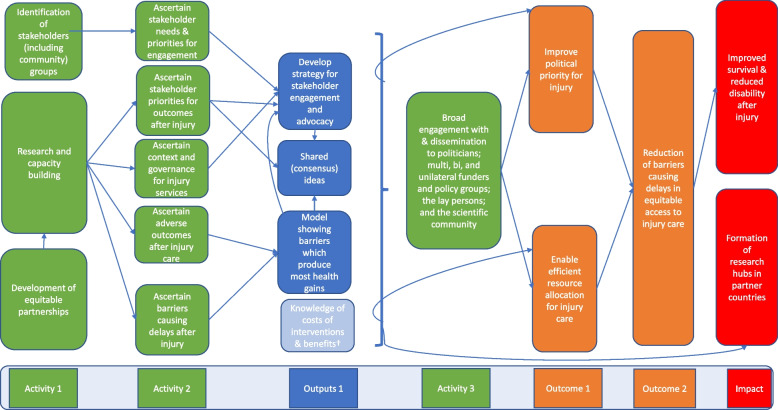
Theory of change diagram for Equi-Injury Project

We expect these activities will result in outcomes of improved political priority and efficient resource allocation for injury care, which will, in turn, result in the outcome of reduction in the barriers to equitable access to quality care after injury. These activities, outputs, and outcomes lead us to the expected impact of improved survival and reduced disability after injury. We also expect that methodological hubs formed across partner countries to deliver the work will be sustained and lead to future impactful research delivery (Fig. 2).

Figure 2 shows activities, outputs, outcomes, and impact anticipated from delivering the project and the connections between these.

### Literature reviews

We will conduct three systematic reviews with narrative synthesis. Protocols for each review will be developed separately, following PRISMA guidelines. We will i. evaluate the existing literature on delays and barriers to accessing injury in each country; ii. assess the extent to which community groups have been involved in strategies or policies to reduce delays and barriers to accessing injury care in LMICs; and iii. determine the lifetime costs of injuries experienced in LMICs.

### Study settings

Data will be collected mainly from two rural and two urban areas or districts in each country, purposively selected to be as representative of the country context as possible whilst ensuring data collection is feasible. Where data are to be collected from healthcare facilities, these will be secondary or tertiary facilities serving injured patients in the rural or urban areas selected for study.

### Work package 1: stakeholder needs for embedding sustainable solutions

The objective of WP 1 is to understand the contexts and needs of key stakeholders (communities and patients; health care workers; and policy makers) for wider involvement in policy for injury care. From the synthesis of this knowledge, the WP will co-create a strategy to facilitate stakeholder involvement and translation of research into policy. This WP answers three research questions: i. What are the levels and mechanisms that stakeholder groups want to be involved in policy and what are the barriers and facilitators to their involvement; ii. are there common priorities across groups that can facilitate engagement of all groups? and iii. what are the policy, finance, and governance contexts for injury care in partner countries?

The first two research questions will be addressed through a series of workshops utilising a modified nominal consensus approach (see Appendix 1). If commonalities are found, we will form multistakeholder groups and facilitate them to work together for the remained of the project to understand the results, develop consensus on barriers to access to quality care after injury to address, and to co-develop solutions. Results will be summarised outputs from the workshops, presented as prioritised lists.

The third research question will be addressed through conducting a desk review of the current context for policy around injury care in each country and by a survey to assess governance contexts for healthcare in LMICs [33], modified for injury care. This modified questionnaire has been evaluated for face validity and feasibility in Rwanda, Ghana, and South Africa [34]. The questionnaire evaluates presence of structures or functions at national, health policy formulation, and implementation levels to assess the whole health system context for injury care.

Results for the governance questionnaire will be described using measure of central tendency and spread for each country in total and by governance survey domains. Results will be disaggregated by respondent type and an exploratory analysis performed to explore differences in score by respondent employment role. Statistical approaches for the comparative analysis will be determined by whether data are normally distributed or not.

### Participants (stakeholders)

Participants will be purposively selected for the workshops to represent the perspectives of the community members, patients who have accessed injury care but who are not under current acute care for their injuries, patients with moderate to severe injuries who have not accessed care for their injuries, healthcare providers, policy makers and civil-servants.

The survey will be completed by 20–30 healthcare workers and policy makers in each country who will be approached to participate by introductory email.

### Work package 2: barriers, quality and priority outcomes

In this WP, we aim to answer three research questions. First, what are the barriers to equitable access to quality care and outcomes are experienced by patients who have suffered injuries? Second, what quality care outcomes are the highest priority to address for patients/community members, healthcare providers, or policy makers/civil servants? Third, is consensus on priority outcomes achievable across stakeholder groups?

#### Settings

The study will be performed in urban and rural areas, utilising 4 hospitals per country and participants from the rural and urban areas which these hospitals serve.

### Research question 1 – barriers to equitable access to quality care

Utilizing data collected through multiple methods as described below, we will assess delays to accessing care, equity of access, and effectiveness, safety, and patient centredness of care received. We will describe the data collection methods, then how those data will be used.

- A. Hospitalised patient data collection.

Data collected will be used to inform IoM domains of effectiveness, equity, timeliness, and patient centredness.

#### Participants and sample size

Data will be collected from injured patients who are admitted to facilities for more than 12 h (used as a proxy for a moderate to severe injury) or died before 12 h. Victims of sexual violence and those not able or willing to give informed consent themselves or via a relative will be excluded.

The sample size for each location in each country is based upon the primary outcome of people who died or were disabled after injury. Based on existing literature, we estimate that 40% of injured participants will die or be disabled [6, 8, 11]. We calculated the sample size as being 1932 per country which leaves 1546 evaluable cases accounting for a 20% drop out based on a combined mortality and disability rate of 40% and a confidence interval of 35–45%.

#### Data collection

Within each hospital, before data collection starts, the pathway of injured patients will be mapped and possible points of entry into the facility and wards documented to ensure all eligible participants are captured.

Data will be collected prospectively from electronic or paper medical records and from interviews with patients or their cares/relatives. Data include demographics (age, sex, and rural or urban location) and socio-economic details (household assets and education), details of injury (mechanism, type, and severity), mode and time taken to travel to the facility, clinical management in the facility, out of pocket payments for care before and during admission, experiential quality of care (based on the in-patient assessment of healthcare survey [I-PAHC]) [35], and discharge details. Patients will be followed up daily whilst in hospital to capture information on outcomes. Questions are based upon a survey tool previously developed and used in a similar study of surgery patients in Sierra Leone [36].

Additionally, patients (or their consenting carers/relatives) will be interviewed by telephone 12 weeks after discharge to determine mortality after discharge and capture information on level of disability (WHO DAS [37], quality of life (EQ-5D [38]), receiving healthcare after discharge and experiences of outpatient care in surviving patients using the out-patient assessment of healthcare (O-PAHC) questionnaire [35].

Data collection forms will be piloted separately in each country prior to roll out to ensure they are contextually appropriate, and adjustments made to ensure contextual understanding. Data quality checks are planned weekly to evaluate the number of respondents, completeness of the data, and response rates.

In Rwanda, data on patient injuries, care received, and costs, will also be acquired from a trauma registry which captures information on over 5000 patients per year admitted with trauma to major trauma hospitals and the national ambulance service (Service D’Aide Medical Urgent [SAMU]) databases which capture the times of people who have been transported to hospital facilities.

#### Outcomes and analyses

Outcomes include time delays to accessing care (timeliness, using questions on time taken to travel to facility), effectiveness of care received (using data on death and disability), and patient centredness of care received (using data from I-PAHC and O-PAHC). Equity will be assessed by comparing outcomes by age, sex, rural or urban location, wealth index (quintiles derived from household assets variables using the principal component analysis method [39]), and education status. Additionally OOP and impoverishing or catastrophic expenditure will be calculated using methods as previously used in Sierra Leone, using national household expenditure data and poverty lines to calculate impoverishing and catastrophic expenditure from local and international sources [36, 40, 41]. These economic outcomes will also be compared using markers of equity as described above.

Analyses will be mainly descriptive. Categorical results will be described as frequencies and percentages; continuous variables will be described using means with standard deviations or medians with interquartile ranges depending on the variables’ distribution. Summaries will be presented for all participants and by sex.

Linear and binary logistical regression analyses (as appropriate) will be used to assess equity by comparing outcomes across groups (as specified above), controlling for confounding variables defined a priori (informed by literature and discussion between authors).

- B. Injured patients who have not sought or received care

To understand delays incurred and outcomes experienced in patients who have not sought care, we will study injured people with moderate to severe injuries who have not sought or accessed secondary or tertiary care. Participants will be identified by contacting local community leaders or healthcare workers who work in the study areas.

#### Data collection

Qualitative interviews will be conducted approximately 12 weeks post-injury and focus on seeking and reaching care. At the end of the interview, the participants will be asked questions from WHO DAS and EQ-5D surveys and questions on variables to inform equity, as detailed above. Interviews will continue until saturation is achieved, to a maximum of 50 patients in each country.

#### Data analysis

Qualitative coding software (NVivo) will be used to facilitate data management, storage, and retrieval in analysis for all qualitative data, including field note/observations. Analysis will be grounded in the data and relevant frameworks. Additionally, combined inductive/deductive analysis will be done to allow capture of emerging themes not covered by the frameworks.

At least two members of the research team will generate codes, discussing coding after each transcript/observation note sheet is coded to ensure all themes are captured and there is agreement between researchers. We will have regular debriefs with the qualitative methods group and the production of summaries of data by country, organised by research questions and emerging themes.

- C. Hospital safety questionnaires

This section of the study will focus on the IoM domain of safety.

#### Participants and sample size

The population will be all clinical staff working in the areas of the facilities identified as receiving patients who have been injured. As guided by the Hospital Survey on Patient Culture Questionnaire tools, we aim for the questionnaire to be completed by at least 50% of clinical staff providing care for injured patients.

#### Data collection

We have created a survey tool based on the Hospital Survey on Patient Culture Questionnaire [42] and an adapted WHO Patient Safety Friendly Hospital Initiative Standards tool [43]. The former focuses largely on attitudes and experiences of safety and the latter evaluates facility safety structures and processes in each of the domains of leadership and management, patient and public involvement, safe evidence-based clinical practice, safe environment, and lifelong learning. Questionnaires will be self-completed by participants.

### Analysis

Analysis will be guided by the manuals for each tool. Scores will be given for whether each individual safety standard was met or not. A total percentage score for each facility will be produced by summing scores for each question, dividing by the maximum possible score if all standards were met and multiplying the result by 100. Scores for the facility will be described in total and disaggregated by domain, sex of the respondent, work-role, and area of work.

- D. Provider competency

Provider competency will be used to assess the knowledge of the healthcare workforce, a key WHO Building Block.

#### Participants and sample size

The study population includes all providers in the study facilities who provide clinical care/make clinical decisions for people with injuries. We aim to conduct this work with at least 50 participants in each country; however, sample size will be dictated by the numbers of relevant providers in the study facilities.

#### Data collection

A vignette tool developed for assessing provider competency to provide care for the injured patients in LMIC settings, developed and piloted as previously described, will be used [44]. Data will be collected on facility type, participant sex, care-provider role, clinical training received, frequency of caring for injuries, and length of time in practice caring for the injured people.

The vignettes are based on four hypothetical scenarios: 1) blunt chest trauma causing tension pneumothorax, 2) penetrating abdominal injury with hypovolaemic shock, 3) severe head injury and 4) isolated lower limb open fracture. These scenarios cover 8 of WHO’s Essential Trauma Care “specific medical goals” [45, 46], and two Lancet Commission on Global Surgery’s “bellwether” surgical procedures [47].

#### Analysis

Mean (SD) score will be described for each individual scenario and all scenarios combined. This will be done for all participants in each country and disaggregated by facility type and location, provider type, training, and length of experience in current role.

- E. Survey of facility readiness to provide care

Facility readiness to provide care will allow assessment of the WHO Building Blocks of infrastructure, human resources, and medicinal products and technologies.

#### Participants

At each facility a member of staff identified by the lead facility clinician as the person most able to answer questions will be invited to participate. In cases where the nominated respondent has no knowledge of availability of a particular item, others will be asked.

#### Data collection

We will use a modified International Assessment of Capacity for Trauma (INTACT) tool to assess facility readiness to provide trauma care [48]. The tool collects data on 40 central elements required to provide trauma care and has been used in multiple LMIC settings, modified to include availability of the essential medicines to manage trauma. Only those facilities that have provided data collection for the hospitalised patient will be sampled. Responses are grouped into five categories of infrastructure, supplies, procedures, equipment and personnel. Surveys will be administered by data collectors who will ask to observe all items that the respondent identifies as present at each facility.

#### Analysis

Items will be assigned a score dependent on whether present and whether observed. Scores will be summed for each facility and represented as a percentage of the maximum achievable score.

Scores will also be disaggregated into the five categories of Infrastructure, supplies, procedures, equipment and personnel.

### Research question 2: quality outcomes with highest priority to address

In this study section, we aim to ascertain which quality outcomes are of the highest priority for stakeholders to address.

#### Participants

People who have participated in the stakeholder workshops described in work package 1, above. Where there is stakeholder attrition, we will replace with a stakeholder with similar characteristics.

#### Data collection

We will use Photo-Voice methodology. Focus group discussion (FGD) will be held with each stakeholder group separately, to orient them to different types of quality outcomes and facilitate discussion amongst group members of their experiences and the quality outcomes which matter to them. Participants will then be requested to capture up to 10 photographs which illustrate their selected priority outcomes. In a second focus group, participant will present the three photos which best capture their priority outcomes and describe why they have selected these. Discussions will be audio-recorded and transcribed for analysis, photographs and group-agreed captions will be collected. If it is not possible to gather enough policy makers/civil servants for a FGD or include Photo Voice with policy makers/civil servants, we will conduct in-depth interviews.

#### Sample size

We aim for between 7 to 10 participants per FGD.

#### Data analysis

Qualitative analysis will be conducted, as described above.

#### Mixed method analysis of WP2

A Convergent Parallel mixed methods approach will be used to synthesise and analyse data collected using all methodologies in WP2 [49]. Findings from each method on whether a barrier is present or absent will be captured in a matrix constructed around relevant frameworks (Three Delays, Quality Healthcare, and WHO Building Blocks), developing a triangulation protocol to produce a convergence coding analysis to display findings emerging from each component of the study. This will be followed by consideration of where there is agreement, partial agreement, silence, or dissonance between findings from different components, and allows emergence of meta-themes which cut across different methods. From this, we will construct a framework for equitable access to quality care after injury, informed by our Main Conceptual Framework.

### Work package 3: injury pathway modelling

In order to understand which elements in the pathway of injury response, care and treatment have the biggest impact on health (based on quality and length of life) and economic outcomes, we will develop an injury pathway model (IPM) which quantifies the associations between barriers and outcomes. The barriers which have the greatest impact on outcomes represent investment priorities. Inequality in health and economic outcomes will also be addressed in the injury pathway model.

The model will represent the pathway of care from the injury, transfer to a health care facility, treatment, rehabilitation, and discharge and will explore variability in components of the pathway, e.g., pre-hospital treatment received, time to calling for transport to the facility, or time to receive definitive treatment in the facility, to determine which are drivers of outcomes. If there are differences in patients outcomes for different time delays, treatments, and such, then we can conclude that reducing this variation, so that everyone receives the ‘best’ care, will improve outcomes. This will be explored for each of the components of the model. Understanding the variation in the outcomes across individuals with identical characteristics, for example injury type and severity, helps to define the optimum patient pathway in terms of maximising health. The model will allow variability to be characterised in terms of the impact on outcomes generated.

Country level trauma data will be combined with data collected from electronic healthcare records (which contains information on costs) and data collected from WP2 to develop and populate the IPM. It will be necessary to supplement this data with external evidence and expert opinion, for example in understanding the long-term costs and consequences of injuries. The injury pathway model must be relevant across all four countries and across all types of injury.

The model will utilise a patient level simulation. This models any variability in the patient pathway by directly using the patient data, rather than aggregate data [50]. Individuals, rather than cohorts of patients progress though the pathway model, generating outcomes for each patient. If heterogeneity is accounted for, any differences in outcomes from identical patients can be assumed to be due to the existing variability. The model may contain data, such as the extrapolated survival, which is uncertain, and therefore it may be necessary to reflect this uncertainty. We will use deterministic sensitivity analysis to explore uncertainty and probabilistic sensitivity analysis if it is feasible to do this within the patient level simulation model.

The outputs of the model will be used to inform prioritisation of interventions which offer the greatest health value net of costs (net health effects), for example, asking the question, *is the time to calling for help a key factor in determining patient outcomes*?

The injury pathways model results will be distilled and presented for non-technical audiences. The delays and health gains shown by the model will be overlaid with the consensus priority outcomes identified in WP2, to show where health gains intersect with priority outcomes shared by all stakeholders.

### Work package 4: consensus priority solutions

In this work package we aim to work with stakeholders to present results, gain consensus on priority barriers to overcome, and develop shared priority solutions to address issues in equitable access to injury care which have been highlighted in previous study WPs.

First, we wish to achieve consensus on the highest priority barriers for community members/patients, healthcare providers, policy makers/civil servants to overcome to improve equitable access to quality care and the potential solutions to address these.

In each country, we will conduct a series of facilitated stakeholder workshops, using the same stakeholders forming the multistakeholder group as described previously. Stakeholder workshops will be conducted with each stakeholder group, separately. Where there is stakeholder attrition, we will replace with a stakeholder with similar characteristics.

We will utilise a nominal group technique – as previously described in Appendix 1—with discussions in roundtable format and plenary followed by voting to create priority lists of first barriers to overcome and second, possible solutions. After the individual stakeholder workshops, the research team will summarise the results and show where there is agreement across stakeholder groups.

We will then convene a facilitated multistakeholder workshop to agree consensus across stakeholders on priority barriers to overcome and possible solutions. Using methods based on the COHESION framework [51], the remainder of the workshop will be utilised to identify priority solutions for each country and outline methods to develop these solutions.

### Work package 5: cross country shared learning

In this final work package, the research team will perform cross-country comparisons to enable knowledge synthesis across multiple settings and further prioritise solutions for testing in future studies. As done in our previous study [15], we will determine if the outcomes from work packages 1–4 are similar or differ between countries and if so, what these can be attributed to. To deliver this work package. Where outcomes are shown quantitatively, differences between countries can be compared using regression analyses with the reasons for these explored using data pertaining to country context from various available resources such as income status, GINI, and governance context score. Where differences are described qualitatively, contextual reasons for these will be explored.

The conclusion of work package 5 will be the final team meeting to agree the shared contexts, needs, outcomes, and solutions and further agree on solutions which should be taken forward to develop in future research studies.

## Discussion

This health system evaluation project is designed to investigate the whole health service journey of injured patients in four diverse LMICs, making it possible to compare the findings across countries and understand which barriers and potential interventions may cross contexts and which might not. Mixed methods are used to provide a deeper understanding of injury care and develop evidence-based interventions within and across partner countries and strong partnership with multiple stakeholders will facilitate utilisation of the results for the co-development of sustainable interventions.

In each country, we will work to embed our findings in policy by involving multiple stakeholder groups in our research. We will also engage with multilateral policy makers from study inception to share results and facilitate global uptake of results.

Patient and public involvement is an essential pillar of this study. Our Community Engagement and Involvement (CEI) has been developed to ensure participation in design and conduct of the study, as well as its dissemination. In addition to being informed of the project, community members will be involved from the beginning of the project, with an aim to foster sustainable involvement in research translation to policy and policy making.

### Supplementary Information

**Additional file: 1 Appendix 1.** Methodologies for workshops.

## Data Availability

No datasets were generated or analysed during the current study.
