# Supplementary material for: Equitable access to quality injury care; Equi-Injury project protocol for prioritizing interventions in four low- or middle-income countries: a mixed method study
Source: BMC Health Serv Res. 2024 Apr 4;24:429. doi: 10.1186/s12913-024-10668-y (PMC10996087; doi:10.1186/s12913-024-10668-y)
Supplement: Supplementary file 1 — Additional file: 1 Appendix 1. Methodologies for workshops. [file 12913_2024_10668_MOESM1_ESM.docx]

***Appendix 1. Methodologies for workshops***

- ***WP1 workshops and In-depth interviews:***

Workshops will be done in rural and urban locations in each country with each stakeholder group, separately, using a nominal group technique. (1) Roundtable and plenary discussions facilitated by trained researchers will be held to capture each group’s priorities for engagement in policy (levels of engagement) as well as mechanisms of engagement, and facilitators and barriers to engagement. Priorities will be captured by the research team during the discussions, de-duplicated, and listed for voting on by the workshop participants to obtain the groups’ top 5 priorities in each discussion area. In recognition that there may be logistical challenges in bringing groups of policy makers together, we will do in-depth interviews with policy makers to capture the same information.

After workshops or interviews are conducted, the top priorities in each discussion area for each stakeholder group will be summarized and listed by the research team. During this process, the research team will remove any responses which, on agreement, appear not to be related to the discussion area. The research team will then discuss and agree where there are common priorities across stakeholder groups for each area.

The common priorities will be presented by the research team to purposively selected representatives from each stakeholder group brought together in a multistakeholder workshop. This facilitated workshop will aim to develop consensus across stakeholders on which areas of policy making they can work together in, which mechanisms they can use to deliver this shared work, and how to capitalise on facilitators and overcome barriers. Outputs from the multistakeholder workshop will be shared priorities in each discussion area agreed at the workshop. These will be summarised by the research team in each country.

In addition to lists of priorities captured from workshops, we will draw on methods and standards developed by RREAL (Rapid Research Evaluation and Appraisal Lab) to capture additional information (2, 3). The RREAL Sheet process supports the systematic collection of data according to the main research questions, rapid summary of findings and identification of emerging themes and to identify convergences and gaps including for further adaption of data collection and analytical processes through regular supportive team reflection. In-depth interviews with policy makers structures using contextually appropriate topic guides develop by local teams. RREAL sheets will also be utilised to capture information from in depth interviews with policy makers.

- ***WP4 workshops***

Lists of priorities at each table and results of voting in each plenary will be captured. The research team will also observe the workshop taking field notes on points of agreement and disagreement. They will map the temporal and relational aspects of the consensus process, the points of conflict, and the implicit and explicit values that informed it. This will allow ascertainment of the extent of agreement (consensus measurement) and resolution of disagreement (consensus development) and enable us to capture the priority outcomes that are recognisable to all stakeholders, and to address points of conflict and value positions.

1. McMillan S, Kelly F, Sav A, Kendall E, King M, Whitty J, et al. The paper we needed at the beginning: How to analyse results from the nominal group technique. Health services and outcomes research methodology. 2014;14:92-108.

2. Vindrola-Padros C, Johnson GA. The use of rapid qualitative research in time-sensitive contexts: Challenges and opportunities. Temporality in qualitative inquiry: Theories, methods and practices. 2021:172-91.

3. Vindrola-Padros C, Johnson GA. Rapid Techniques in Qualitative Research: A Critical Review of the Literature. Qualitative Health Research. 2020;30(10):1596-604.
